# Development of multivariate NTCP models for radiation-induced hypothyroidism: a comparative analysis

**DOI:** 10.1186/1748-717X-7-224

**Published:** 2012-12-27

**Authors:** Laura Cella, Raffaele Liuzzi, Manuel Conson, Vittoria D’Avino, Marco Salvatore, Roberto Pacelli

**Affiliations:** 1 Institute of Biostructures and Bioimaging, National Council of Research (CNR), Naples, Italy; 2 Department of Diagnostic Imaging and Radiation Oncology, Federico II University School of Medicine, Naples, Italy

**Keywords:** NTCP modeling, Radiotherapy, Hypothyroidism, Bootstrapping

## Abstract

**Background:**

Hypothyroidism is a frequent late side effect of radiation therapy of the cervical region. Purpose of this work is to develop multivariate normal tissue complication probability (NTCP) models for radiation-induced hypothyroidism (RHT) and to compare them with already existing NTCP models for RHT.

**Methods:**

Fifty-three patients treated with sequential chemo-radiotherapy for Hodgkin’s lymphoma (HL) were retrospectively reviewed for RHT events. Clinical information along with thyroid gland dose distribution parameters were collected and their correlation to RHT was analyzed by Spearman’s rank correlation coefficient (Rs). Multivariate logistic regression method using resampling methods (bootstrapping) was applied to select model order and parameters for NTCP modeling. Model performance was evaluated through the area under the receiver operating characteristic curve (AUC). Models were tested against external published data on RHT and compared with other published NTCP models.

**Results:**

If we express the thyroid volume exceeding X Gy as a percentage (V_x_(%)), a two-variable NTCP model including V_30_(%) and gender resulted to be the optimal predictive model for RHT (Rs = 0.615, p < 0.001. AUC = 0.87). Conversely, if absolute thyroid volume exceeding X Gy (V_x_(cc)) was analyzed, an NTCP model based on 3 variables including V_30_(cc), thyroid gland volume and gender was selected as the most predictive model (Rs = 0.630, p < 0.001. AUC = 0.85). The three-variable model performs better when tested on an external cohort characterized by large inter-individuals variation in thyroid volumes (AUC = 0.914, 95% CI 0.760–0.984). A comparable performance was found between our model and that proposed in the literature based on thyroid gland mean dose and volume (p = 0.264).

**Conclusions:**

The absolute volume of thyroid gland exceeding 30 Gy in combination with thyroid gland volume and gender provide an NTCP model for RHT with improved prediction capability not only within our patient population but also in an external cohort.

## Background

Radiation-induced hypothyroidism (RHT) is a frequent side effect after therapeutic irradiation of the cervical region and it has been described in patients undergoing radiation therapy (RT) for different neoplasms such as lymphoma, head-and-neck cancer and breast cancer [1-3].

The amelioration of life span expectations of cancer patients requires the maximum possible effort to reduce iatrogenic diseases like RHT. The evolution of radiation therapy technology has enhanced the ability to adapt RT techniques to the individual patient. However, in order to establish tailored strategies for a risk-adapted RT, it is essential to identify specific clinical and dosimetric parameters that are involved in the process of modeling normal tissue complication probability (NTCP). Input parameters have been recognized to be among the most critical features of an effective NTCP model [4]. Models that take into account relationships among different patient-related and dosimetric factors may offer a powerful approach to the optimization of risk ascertainment for many different endpoints [5]. As a consequence, data-driven multivariate modeling of NTCP [6] is increasingly being used unlike traditional NTCP models that only involve dose distribution parameters of a specific organ at risk like the Lyman-Kutcher-Burman model.

Recently, a multivariate NTCP model for RHT based on mean thyroid dose and thyroid volume was developed by Boomsma et al [7] in patients treated for head-and-neck cancer. A thyroid volume effect in RHT development, following RT of breast cancer, was also emphasized in a case-control study where the absolute volume receiving more than 30 Gy was recognized as a critical factor for hypothyroidism development [8]. In a previous work [9] on RHT in Hodgkin’s lymphoma (HL) patients, after conventional multivariate analysis method, the percentage of thyroid volume exceeding 30 Gy (V_30_(%)) was found to be the only predictor of RHT. All the above mentioned results, although similar, are not coincident and seem to suggest different prognostic variables for RHT among patients from different populations.

In this framework, the present report expands on the potential of building an effective multivariate NTCP model for RHT and extends the complexity of the analysis in order to evaluate if general information on RHT risk assessment may be extrapolated regardless of the cohort of patients on which the model is built on. To this end, NTCP modelling exercises were performed using bootstrapping together with validation and performance comparisons on different patients cohorts evaluated for RHT using data from the literature [7-9].

## Methods

### Patient dataset

Data on 61 consecutive patients with HL undergoing post-chemotherapy supradiaphragmatic involved-field radiation therapy at the Radiation Oncology Department of the University “Federico II” of Naples were retrospectively reviewed for RHT events. Selection criteria included the patients informed consent, availability of thyroid hormones serum data before chemotherapy, after chemotherapy, and, periodically, after RT as well as the availability of treatment planning data. Blood levels of thyroid stimulating hormone (TSH), free triiodo-thyronine (FT3), free thyroxine (FT4), thyroglobulin antibody (ATG) were evaluated. The study was approved by the ethics committee of our institution. A diagnosis of RHT (event) was based on TSH value greater than the maximum value of laboratory range and/or FT3 and/or FT4 values lower than the minimum value of laboratory range, whether any symptom was present or not (subclinical or clinical RHT). Eight patients (13.1%) had hypothyroidism before treatment, and were consequently excluded from further evaluation. General patient characteristics are given in Table 1. Twenty-two out of 53 patients (41.5%) developed laboratory evidence of hypothyroidism at a median follow-up of 32 months (range 6–99) after the end of radiation treatment [9].

**Table 1:** Patient, disease and treatment characteristics

*Median age (years)*	27.5 (14–70)	
*Median thyroid volume (cc)*	13.7 (6.7–44.0)	
*Gender*	***N***	***%***
Male	25	47.2
Female	28	52.8
*Histology*		
Nodular sclerosis	38	71.7
Mixed cellularity	10	18.9
Lymphocyte-rich-classical	5	9.4
*Stage*		
I-II	42	79.2
III-IV	11	20.8
*Radiotherapy dose delivered*		
30 Gy	23	43.4
32 Gy	25	47.2
36 Gy	5	9.4
*Chemotherapy regimen*		
ABVD	15	28.3
VEBEP	38	71.7

All patients were treated with full 3D CT based radiation treatment planning as described in detail in a previous publication [10]. In short, three-dimensional conformal plans were generated using a commercial treatment planning system (XiO, Elekta CMS. St Louis. MO) and the convolution dose calculation algorithm, appropriate in the presence of heterogeneous tissues, was applied. RT was administered using 6 -20 MV photon beams from a linear accelerator with anteroposterior-posteroanterior fields. A total median dose of 32 Gy (range 30–36) in 20 daily fractions of 1.5–1.8 Gy was planned. For all patients, the thyroid gland was retrospectively delineated on purpose on the planning CT-images by the same radiation oncologist (M.C.). The thyroid gland volume, the minimum (D_min_), maximum (D_max_) and mean doses (D_mean_), the absolute volume of thyroid and the percentage of thyroid volume exceeding 10, 20 and 30 Gy (V_x_(cc) and V_x_(%), respectively) were calculated from the dose volume histograms. In addition, the “residual X Gy thyroid volume”, defined as the difference between the thyroid gland volume and Vx (cc), was calculated.

### Statistical modeling

Dosimetric parameters of the thyroid gland along with patient clinical information (thyroid gland volume, age, gender, chemotherapy, and clinical stage) were included in the analysis. Univariate logistic analysis for each variable was performed using the Spearman’s rank correlation (Rs) coefficient to assess inter-variable correlation and correlation with RHT risk.

We separately analyzed two sets of candidate predictors: set 1 includes the clinical variables, plus D_min,_ D_max,_ D_mean_ and V_x_(%), and set 2 includes the same variables as set 1 but V_x_ was expressed as absolute volume, V_x_ (cc).

To identify combinations of variables that were likely to be most predictive of RHT, we used automated logistic regression with bootstrap technique for variable selection and bootstrap resampling to test selection stability [6]. The logistic regression model is defined as

(1) $$\mathrm{NTCP}\;=\;\frac{1}{1\;+\;e^{-g\left(x\right)}}$$

with

(2) $$g\left(x\right)\;=\;\beta_{0}\;+\;\beta_{0}x_{1}\;+\;\beta_{1}x_{1}\;+\;\ldots \beta_{n}x_{n}$$

Where *x*_*1*_*, x*_*2*_*.…. x*_*n*_ represent different input variables and *β*_*0*_*, β*_*1*_*.…. β*_*n*_ are the corresponding regression coefficients.

In order to avoid overfitting, when the Rs coefficient between two variables was greater than 0.85 we excluded the one with the lowest correlation with RHT [11] from the subsequent multivariate analysis.

Data analysis was performed by an open source available package (Dose Response Explorer System [12]) for combined modeling of multiple dosimetric parameters and clinical factors using multi-term regression modeling. In summary, the modeling process consists of a two-step process. In a first step, the model size (number of variables significantly predictive) is estimated by bootstrapping and in the second step regression coefficients are estimated using forward selection on multiple bootstrap samples, the most frequent model being the optimal one. Model predictive power is quantified using Rs correlation coefficient while the area under the receiver operating characteristic curve (AUC) was used to evaluate the discriminating ability of model fits.

Subsequently, the obtained NTCP models were validated against an independent external cohort. To this end, data on RHT in breast cancer patients with irradiated supraclavicular lymph nodes were taken from the literature [8]. For comparison purpose, we also evaluated the NTCP model for RHT proposed by Boomsma et al [7] that is based on thyroid gland mean dose and volume. Model comparison was performed using a z test on the AUC of receiver operating characteristic (ROC) curves. A p value less than 0.05 was considered statistically significant. Statistics was performed using MedCalc (MedCalc, Mariakerke, Belgium).

## Results and discussion

### Models

**Figure 1:**
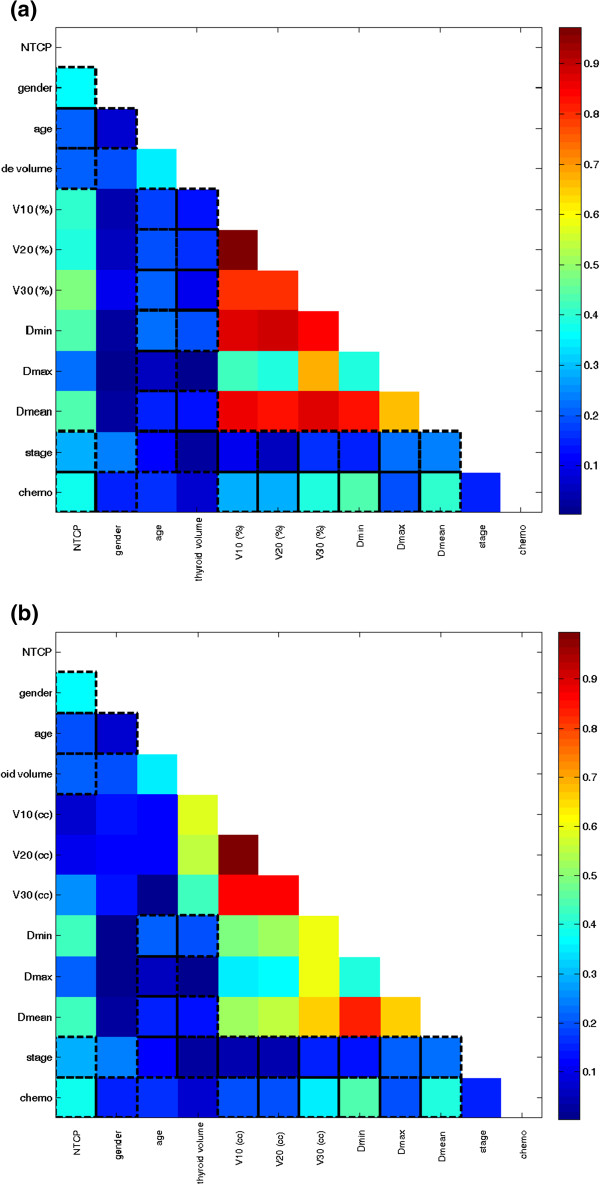
**The cross-correlation matrixes for the variables belonging to set 1 (a) and to set 2 (b).** The colorbar represents the Spearman’s rank correlation coefficient value. NTCP: normal tissue complication probability , Vx (%):percentage of thyroid volume exceeding X Gy; Vx (cc): absolute thyroid volume exceeding X Gy.

The cross-correlation matrixes for the variables belonging to set 1 and set 2, respectively, are shown in Figure 1a-b. For both set of variables, a strong multiple correlation (i.e. Rs > 0.85) between dosimetric parameters was found. After applying the selection criteria to avoid overfitting to set 1, V_30_ (%) and D_max_ resulted to be the dosimetric parameters that should be included in the multivariate analysis along with clinical variables. Similarly, for set 2, V_30_ (cc), D_max_ e D_mean_ were selected along with clinical variables.

**Figure 2:**
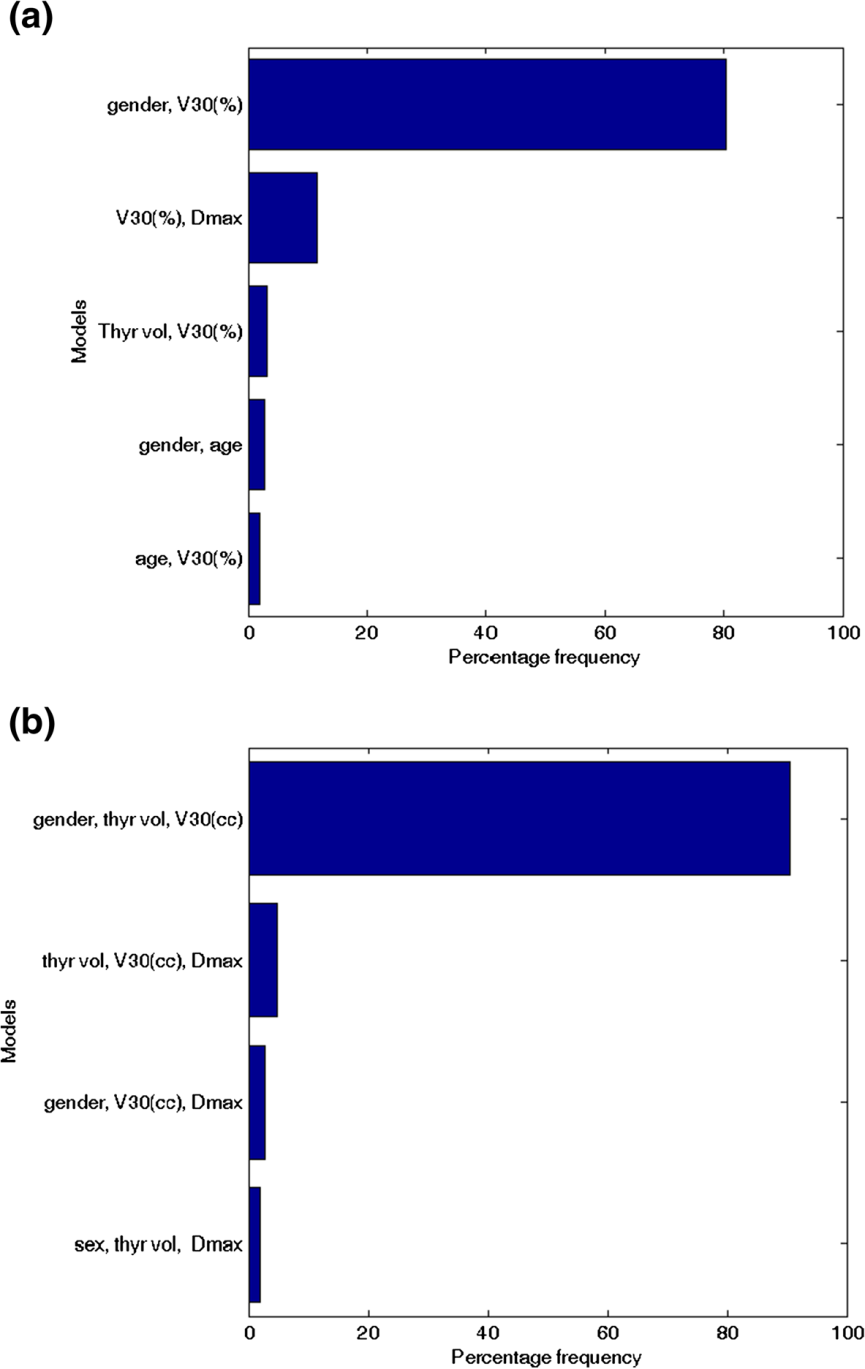
**The five most frequently selected models by bootstrap sampling technique: (a) variable set 1; (b) variable set 2.** NTCP: normal tissue complication probability , Vx (%): percentage of thyroid volume exceeding X Gy; Vx (cc): absolute thyroid volume exceeding X Gy.

In set 1, a two-variable model was suggested as the optimal order by bootstrap method. Figure 2a shows the five most frequently selected models within the bootstrapped subpopulations. The optimal model (Rs = 0.615, p < 0.001) includes gender (female =0, male = 1) and V_30_(%) (model 1). The best-fitted regression coefficients are given in Table 2. According to this model, the risk of RHT increases as V_30_(%) increases, and it is higher for female patients.

**Table 2:** Best-fitted regression coefficients and 95% confidence intervals for model 1 and model 2

**Parameter**	**Estimated coefficient**	**StdError**	**p-value**
*Model 1*			
gender	−2.32	0.83	0.0062
V_30_(%)	0.038	0.01	0.0009
constant	−1.83		
*Model 2*			
gender	−2.21	0.85	0.0110
V_30_(cc)	0.26	0.09	0.0021
thyroid volume (cc)	−0.27	0.11	0.0140
constant	1.94		

**Figure 3:**
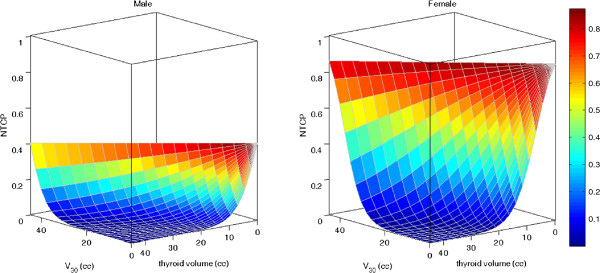
Model 2 NTCP surfaces for males and females as a function of V30(cc) and thyroid volume (cc).

Conversely, in set 2, a three-variable model was suggested as the optimal order by bootstrap method. Figure 2b shows the five most frequently selected models within the bootstrapped subpopulations. The optimal model (Rs = 0.630, p < 0.001) includes gender, V_30_(cc) and thyroid gland volume (model 2). The best-fitted regression coefficients are given in Table 2. As for model 1, the risk of RHT increases as V_30_(cc) increases and it is higher for female patients; in addition the risk decreases with larger volume of thyroid gland. Model 2 NTCP surfaces for males and females are represented in Figure 3.

Actually, we can consider the above model 1 and model 2 as equivalent models being V_30_(%) the ratio of V_30_(cc) to thyroid gland volume. In our previous work [9] we already found that thyroid V_30_(%) predicts the risk of developing RHT. However other groups [7,8] have shown a thyroid gland volume effect in RHT development: the risk increases with smaller thyroid gland volume. For this reason, in this work we separately analyze the V_x_ parameters as percentages and as absolute volumes.

It is interesting to note that both our NTCP models include gender. This result is in agreement with the meta-analysis by Vogelius et al [13] who identified gender, together with race and surgery of the neck, to be as a significant prognostic clinical variable in RHT development.

### Models’ comparison and validation

**Figure 4:**
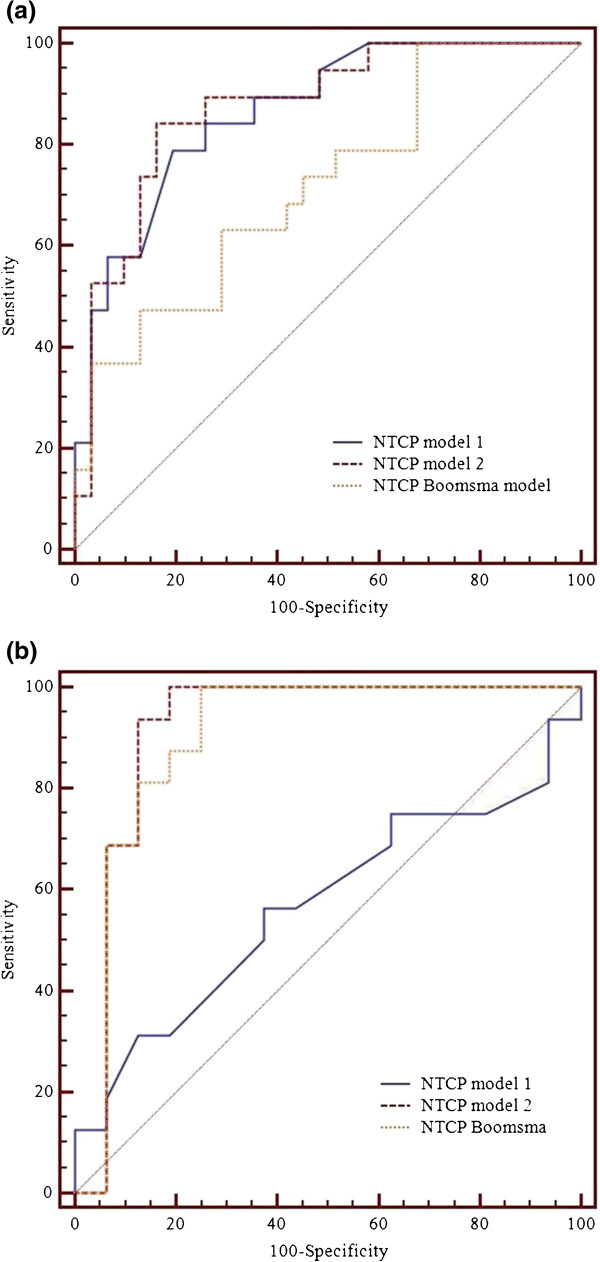
**ROC curves for model 1, model 2, and Boomsma model**[7]**: (a) on Hodgkin’s lymphoma dataset (b) on external breast cancer dataset**[8]**.**

The obtained models were then compared by using the AUC (Table 3) of the ROC curves depicted in Figure 4a-b. As expected, no difference in performance was found between model 1 and model 2 (p = 0.76) for our cohort of patients (Figure 4a).

**Table 3:** **Area under the receiver operating characteristic curve (AUC) and 95% confidence intervals for all the models applied on our Hodgkin’s lymphoma (HL) dataset and on an external breast cancer dataset**[8]

		**AUC**	**95% CI**	**p value**
*HL dataset*				
	Model 1	0.865	0.793–0.945	
	Model 2	0.874	0.750–0.951	0.760^*^
	Boomsma model [7]	0.718	0.573–0.836	0.044^*^
				0.023^§^
*External dataset*				
	Model 1	0.568	0.382–0.741	
	Model 2	0.914	0.760–0.984	0.005^*^
	Boomsma model [7]	0.898	0.740–0.977	0.009^*^
				0.264^§^

Significance level was obtained using z test.

^*^ evaluated respect to model 1.

^§^ evaluated respect to model 2.

Applying model 1 and model 2 to the external case-control cohort of breast cancer patients, we have obtained the ROC curves showed in Figure 4b. In this case, model 1 fails to predict RHT (AUC = 0.568, 95% CI 0.328-0.741) while model 2 has a high performance (AUC = 0.914, 95% CI 0.768–0.984). This result can be ascribed to the fact that, unlike our patients, the external cohort is characterized by large inter-individual and inter-group variations in thyroid volumes. Therefore model 2, where V_30_ is expressed as absolute volume coupled with the thyroid volume, results to be more effective in RHT prediction.

Subsequently, we have analyzed the Boomsma NTCP model for RHT. It should be noted that these authors reported an AUC of 0.85 (95% CI 0.78–0.92) on their head-and-neck cancer patient dataset. The Boomsma model and model 1 and model 2 performances are not statistically different (p = 0.67) when each is evaluated on its own internal data set.

The ROC curves generated applying the Boomsma model on our HL dataset and on the breast cancer dataset are shown in Figure 4a and 4b, respectively.

On our cohort of patients, the performance of Boomsma NTCP model resulted statistically lower than that of model 1 or model 2 (p < 0.05). Conversely, on validation breast cancer cohort model 2 and Boomsma model have comparably high performance (p = 0.26).

Based on the AUC analysis, both model 2 and Boomsma model seem to be successfully applicable to predict RHT also on a different population.

The difference between the above models relies on the use of V_30_ (cc) and gender for model 2 and on the use of D_mean_ for Boomsma model, while the thyroid gland volume is a common variable. The different selection of dosimetric variables may be ascribed to the relatively high uniform thyroid dose distribution in a head-and-neck cancer cohort (where up to 70 Gy are prescribed with a V30(cc) probably equal to the thyroid gland volume) compared with thyroid dose distribution in our Hodgkin lymphoma patients treated with a median dose of 32 Gy [14].

Besides the prediction performance, we believe that a model that also considers gender could be advantageous being the estimated rate of hypothyroidism in the general population higher in women than in men [15]. In addition, to explain higher susceptibility of women to RHT, it has been assumed that RT could work as a multiplicative factor that increases the baseline risk of the general population [13]. This could justify the comparable performance of model 2 and Boomsma model when applied on a uniform female cohort as the breast cancer patient dataset, while a lower performance of Boomsam model is observed when it is applied on HL patients where female and male are almost homogenously represented.

In treatment planning optimization procedures, the separate use of thyroid gland volume along with a dosimetric parameter (V_30_(cc) or D_mean_) is not easily tunable. In this framework, the “residual 30 Gy thyroid volume” defined as the difference between the thyroid gland volume and V_30_(cc) may be easier to use. From our HL data, the “residual 30 Gy thyroid volume” was found to be a significant predictor of RHT as well (Rs = 0.56). The median “residual 30 Gy thyroid volume” of patients with RHT was 0.2 cc (range 0.0-15.6 cc) in contrast to a median value of 9.4 cc (range 0.0-31.2 cc) for those without RHT. From ROC analyses we have estimated a cutoff volume equal to 7 cc (AUC = 0.81, 95% CI 0.720-0.904) for the “residual 30 Gy thyroid volume” as a critical value above which there is a high probability for the thyroid to maintain its functionality. This result is in agreement with the work by Johansen et al [8] where a median residual 30 Gy thyroid volume of 5 cc was found in patients who developed RHT in contrast to a median value of 11 cc in patients who did not develop RHT.

## Conclusions

In this study we have developed a multivariate NTCP model for RHT based on dosimetric and clinical variables: the absolute volume of thyroid gland exceeding 30 Gy, thyroid gland volume and gender. This three-variable model provides an improved prediction capability not only within our patient population but also in an external validation cohort. In addition, we have found a cutoff “residual 30 Gy volume” for thyroid gland that should be considered in the treatment planning procedure in order to maintain the gland functionality.

## Abbreviations

ATG: Thyroglobulin antibody; AUC: Area under the curve; CI: Confidence interval; FT3: Free triiodo-thyronine; FT4: Free thyroxine; HL: Hodgkin’s lymphoma; NTCP: Normal tissue complication probability; RHT: Radiation-induced hypothyroidism; ROC: Receiver operator characteristic; Rs: Spearman’s rank correlation; RT: Radiation therapy; TSH: Thyroid stimulating hormone.

## Competing interests

The authors declare that they have no competing interests.

## Authors’ contributions

LC, RL and RP conceived and designed the study. MC, MS and RP reviewed patient data. LC, RL, VDA performed the statistical modeling and analysed the data. All authors participated in drafting and revising the manuscript. All authors have given their final approval of the manuscript.
